# Postpartum urinary incontinence and birth outcomes as a result of the pushing technique: a systematic review and meta-analysis

**DOI:** 10.1007/s00192-021-05058-5

**Published:** 2022-02-01

**Authors:** Katsuko Shinozaki, Maiko Suto, Erika Ota, Hiromi Eto, Shigeko Horiuchi

**Affiliations:** 1 International University of Health and Welfare, 2-6-16 Momochiham, Sawara-ku, Fukuoka-city, 814-0001 Japan; 2 National Center for Child Health and Development, Tokyo, Japan; 3 Tokyo Foundation for Policy Research, Tokyo, Japan; 4 Nagasaki University, Nagasaki, Japan; 5 St Luke’s International University, Tokyo, Japan

**Keywords:** Pushing technique, Bearing down, Second stage of labor, Urinary incontinence, Perineal lacerations, Delivery positions

## Abstract

**Introduction and hypothesis:**

Directed pushing while using the Valsalva maneuver is shown to lead to bladder neck descent, especially in women with urinary incontinence (UI). There is insufficient evidence about the benefits or adverse effects between the pushing technique during the second stage of labor and urinary incontinence postpartum. The objective of this study was to evaluate the effects of the pushing technique for women during labor on postpartum UI and birth outcomes.

**Methods:**

Scientific databases were searched for studies relating to postpartum urinary incontinence and birth outcomes when the pushing technique was used from 1986 until 2020. RCTs that assessed healthy primiparas who used the pushing technique in the second stage of labor were included. In accordance with Cochrane Handbook guidelines, risk of bias was assessed and meta-analyzed. Certainty of evidence was assessed using the GRADE approach.

**Results:**

Seventeen RCTs (4606 primiparas) were included. The change in UI scores from baseline to postpartum was significantly lower as a result of spontaneous pushing (two studies; 867 primiparas; standardized mean difference: SMD –0.18, 95% CI –0.31 to –0.04). Although women were in the recumbent position during the second stage, directed pushing group showed a significantly shorter labor by 21.39 min compared with the spontaneous pushing group: there was no significant difference in the duration of the second stage of labor between groups.

**Conclusions:**

Primiparas who were in the upright position and who experienced spontaneous pushing during the second stage of labor could reduce their UI score from baseline to postpartum.

## Introduction

Pregnancy and childbirth are factors that contribute to pelvic floor disfunction (PFD). Thirty percent of postpartum women experience urinary incontinence [1, 2]. Most care for postnatal urinary incontinence is focused on treatment for PFD; however, pelvic floor muscles do not work alone, but work in cooperation with other muscles.

The whole abdominal cavity has been shown to work in conjunction with the pelvic floor muscles [3]. The abdominal cavity is referred to as the inner unit with the diaphragm on the upper side, the transversus abdominis muscle on the side, the multifidus muscle on the back side, and the pelvic floor muscles on the bottom side. All muscles are connected to each other [3–5].

Diaphragmatic motion is related to the contraction of the pelvic floor muscles [5]. The diaphragm is the muscle responsible for breathing, rising with exhalation and descending with inspiration. The pelvic floor muscles also move in conjunction with this movement, descending with expiration and rising with inspiration. Spontaneous pushing during labor involves natural exhalation within a short time frame of 6 s [6, 7] , whereas directed pushing involves consciously applying strong abdominal pressure and bearing down > 10 s or as long as a contraction continues [8, 9]. Generally, the average time for the second stage of labor is 1 or 2 h; during this time, women continue using either pushing technique [10].

If women continue to experience strong abdominal pressure such as that experienced during directed pushing for a long period of time, PFMs will loosen. Loosened PFMs cannot support pelvic organs such as the bladder, which leads to bladder descent and subsequently urinary incontinence [11]. Direct pushing like that involved when using the Valsalva maneuver is considered to cause damage to the PFMs.

In this study, we aimed to systematically review whether the pushing technique used by women in the second stage of labor affects postpartum urinary incontinence and birth outcomes.

## Materials and methods

This study was registered in the International Prospective Register of Systematic reviews (PROSPERO), with registration number CRD42017070826.

### Search and selection of studies

We searched CINAHL, the Cochrane Library, EMBASE, MEDLINE, and PubMed on January 29, 2021, for articles related to postpartum urinary incontinence and birth outcomes when the pushing technique was used. We followed the Cochrane Handbook and Preferred Reporting Items for Systematic Reviews and Meta-Analyses (PRISMA) guidelines for reporting the review results [12]. There were no restrictions on the date/time, language, document type, and publication status. The keywords were identified from experts’ opinions, literature review, controlled vocabulary [CINAHL Headings; Medical Subject Headings (MeSH); Excerpta Medica Tree (EMTREE)], and reviewing the primary search results. We used Eisinga’s animal search filter to exclude non-human search results in EMBASE. Because of the poor reporting of outcomes in medical research, we did not limit the search in any way so as to enable us to obtain all search outcome results. The search strategies were developed with the assistance of a medical information specialist.

### Eligibility criteria

1. Participants: These eligible women were primiparas at term during labor with a vertex singleton alive fetus and absence of complications. We excluded multiparous women and those with past history of urinary incontinence, anal incontinence, and pelvic organ prolapse and those who had caesarean section.
2. Interventions: Spontaneous pushing is defined as the naturally exhalation method where the woman pushes when she feels the urge. It includes delayed pushing and uncoached pushing.
3. Control: Directed pushing is defined as when women take a deep breath and hold it during the peak of contraction and then bear down and push for 10 s; this is repeated for the duration of the contraction. It includes immediate pushing, coached pushing, the Valsalva maneuver (pushing), and the breath-holding method.
4. Search strategy: This strategy was designed according to Population, Intervention, Comparison, Outcomes, and Studies (PICOS) criteria as shown in Table 1. The types of studies included were individual and cluster randomized controlled trials (RCTs). Study design included RCTs.
5. The primary outcome was urinary incontinence. The secondary outcomes were perineal related, such as no suturing of the perineum, third- or fourth-degree laceration and episiotomy, and duration of the second stage of labor.

**Table 1:** PICOS criteria to guide the systematic review

Population	Primiparous
Intervention	Spontaneous pushing, delayed pushing, or uncoached pushing
Comparison	Directed pushing, immediate pushing, or coached pushing, Valsalva maneuver(pushing) Take a deep breath, hold it and push, or early pushing
Outcomes	Primary: urinary incontinence at postpartum Secondary: No perineal laceration (intact perineum), Third- or fourth-degree laceration Episiotomy, Length of second stage of labor
Study design	Randomized controlled trial; RCT

### Study selection

We used Rayyan (http://rayyan.qcri.org), a free web application for speeding up the selection process of studies for inclusion within this systematic review. The search results were de-duplicated using EndNote χ6 and sent to two researchers for screening and confirmation. Two authors (KS, MS) independently screened all titles and abstracts so that non-eligible trials were excluded. When the two authors disagreed about study inclusion, other authors (EO, HE, SH) were consulted to obtain a consensus decision. All selected eligible studies were included in the present systematic review, and the appropriate data for statistical synthesis were included for the meta-analysis using Review Manager (RevMan) 5.4.1 (https://community.cochrane.org/help/tools-and-software/revman-5/revman-5-download).

### Data analysis

We extracted both continuous and dichotomous data using Rev Man 5.4.1.

For continuous data, we calculated the mean difference with 95% confidence intervals (CI) for each study using the fixed-effect model. For dichotomous data, we calculated the risk ratio (RR) with 95% CI of each study using the fixed-effect model. We assessed heterogeneity using the ^2^ Chi^2^ and I^2^ tests. If multiple comparisons were made, only the groups that matched the intervention were selected and included in the statistical analysis.

Certainty of evidence was assessed using the GRADE approach [13]. The GRADE approach consisted of five domains, namely, study limitation (risk of bias), consistency of effect, indirectness, imprecision, and publication bias. A summary of findings included: change in urinary scores, urodynamic stress incontinence, no suturing, episiotomy, third- or fourth-degree laceration, and duration of the second stage of labor. The quality of the body of evidence was evaluated at four levels, namely, “high,” “moderate,” “low,” and “very low.”

### Assessing risk of bias

Risk of bias was assessed by using the Cochrane Handbook risk of bias tool [12]. This assessment included seven items, namely, random sequence generation (selection bias), allocation concealment (selection bias), blinding of participants and personnel (performance bias), blinding of outcome assessment (detection bias), incomplete outcome data (attrition bias), selective reporting (reporting bias), and other biases.

Three authors (KS, MS, EO) independently judged the risk of bias of each included study. For any disagreements, the authors discussed the study until a consensus was made.

## Results

### Search results

**Fig. 1:**
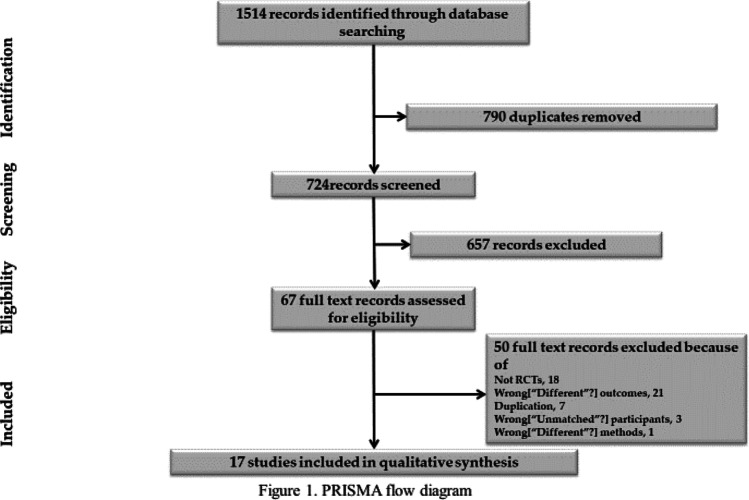
PRISMA flow diagram

After duplicate studies were removed, 724 records were screened overall. A total of 658 records were excluded from the reviewed records because of the absence of RCTs, differences in the participants or interventions, or no outcomes data, as determined by Rayyan. From the remaining 67 appropriate studies, 51 were excluded because of the absence of RCTs, wrong participants, incorrect methods, wrong outcomes, and duplications. Seventeen RCTs with 4606 primiparas (2324 primiparas in the spontaneous pushing group and 2282 primiparas in the directed pushing group) met eligibility criteria for inclusion in our study. The selection process of studies is shown in the PRISMA flow diagram (Fig. 1).

### Characteristics of included studies

The characteristics of the individual studies included are shown in Table 2. An RCT design applied included studies [14–30]. All studies characterized their participants as low-risk primiparas women at term with a singleton fetus and vertex presentation. The exclusion criteria were multiparous women and women with pregnancy complications [14–30].

**Table 2:** Characteristics of individual studies

Authors	Year	Location	Study type	Sample size	Intervention group (spontaneous pushing)	Control group (directed pushing)	Outcome reported	Results	
								Spontaneous pushing	Directed pushing
Ahmadi Z et al. [14]	2017	Iran	RCT 166	Breathing technique group: 83 Valsalva maneuver group: 83	Breathing techniques (case group): The women were asked to take two deep abdominal breaths and push for 4–5 s with an open mouth while controlling exhalation and then resume the process for the next push as trained	Control group (Valsalva maneuver): pushing was carried out according to delivery room routine by holding the breath	No trauma Perineum lacelation (I–III) Episiotomy	Perineal outcomes n = 83 no trauma 34(41%) Perineal laceration: 12 (14.3%) 1st degree 8 (69%) 2nd degree 4 (31%) 3rd degree 0 Anterior laceration: 22 (26.5%) Episiotomy 15 (18.2%)	Perineal outcomes n = 83 no trauma 16(19.3%) Perineal laceration: 29 (34.9%) 1st degree 12 (41%) 2nd degree 16 (56%) 3rd degree 1 (3%) Anterior laceration: 17 (20.5%) Episiotomy 21 (25.3%)
Bloom et al. [15]	2006	US	RCT 320	Uncoached group n = 157 Coached group n = 163	Uncoached group Step 1: Head of bed up 30° Step 2: Position patient, as she desires, on her back or either side Step 3: The patient should be told simply to "do whatever the patient feels the urge to while in bed"	Coached group Step 1: Head to be placed up 30° Step 2: Position patient, as she desires, on her back or either side Step 3: Coach patient to pull back on both knees and tuck her chin while the provider or partner supports the legs Step 4: Coach patient to take a deep breath and hold it during the peak of a contraction then bear down and push for 10 s; repeat this as long as the contraction continues	Length of second stage (min) Perineal laceration Episiotomy	Perineal outcomes n = 157 No or first degree 110 51%) 2nd degree 32 (20%) 3rd degree 13 (8%) 4th degree 2 (1%) Episiotomy 32 (20%)	Perineal outcomes n = 163 No or first degree 105 (45%) 2nd degree 40 (24%) 3rd degree 12 (7%) 4th degree 6 (4%) Episiotomy 42 (26%)
Hansen et al. [16]	2002	US	RCT 129	Pushing group n = 65 Rest group n = 64	Rest group: Women in the experimental group began a period of rest and descent at the time of complete dilatation and continued until the head was seen at the introitus, or after 120 min. These women were encouraged not to push. Visualizing the head at the introitus was determined by sightly separating the vulva with two fingers during a contraction, with the woman's legs apart. The introitus was examined in this manner every 30 min, or sooner if signs of imminent delivery occurred. In the experimental group, the nurse determined the station and position at the onset of pushing after the period of rest and decent.	In the control group women were encouraged to begin pushing as soon as they were found to be completely dilated	Length of second stage (min)	Length of second stage of labor Rest group: n = 64 Mean (min) 171 (56.76)	Length of second stage of labor Pushing group: n = 65 Mean (min) 75.77 (41.33)
Kelly et al. [17]	2010	US	RCT 59	Delayed: 26 Immediate: 33	Delayed pushing group: After dilatation of 10 cm was reached, the VAS was completed, and subjects randomized were told to rest for 90 min or until they felt an uncontrollable urge to push (whichever came first) before they began pushing. A 90-min rest period was selected based on a review of recent publications on delayed pushing, which found that the delay interval ranged from 60 to 120 min. Pushing was allowed to begin before and after the 90-min rest period if the subjects felt an unconfortable urge to push or if the fetal head was visualized at the intoroitus. Instructions for pushing were provided in the same manner as for the immediate pushing group, subjects used a variety of positions during second stage of labor	Immediate pushing group: After dilatation of 10 cm was reached, the subjects were then directed to begin pushing. The subjects were instructed to push three to four times during each contruction by bearing down in a manner similar to the bearing-down effort used to have a bowel movement. No provider counting during pushing occurred. Both open and glottis methods were used, depending on subject's preference and effectiveness of pushing effort as determined by progressive fetal decent. Subjects used a variety of positions during the second stage of labor	Length of second stage of labor perineal lacelation (3rd degree, 4th degree)	Delayed pushing n = 26 Perineal laceration 3rd degree: 1 (7%) 4th degree: 0 (0%) Length of second stage of labor Delayed pushing n = 16 Mean (min): 117.6 (12.1)	Immediate pushing n = 33 Perineal laceration 3rd degree: 2 (8%) 4th degree: 0 (0%) Length of second stage of labor Immediate pushing n = 28 Mean (min): 87.1 (8.6)
Low et al. [18]	2013	US	RCT 73	Spontaneous pushing: 34 Direct pushing: 39	Spontaneous pushing: with instruction provided prenatally via a standardized training video. This method included instructing the woman to follow her bodily sensation and push as she felt the urge	Directed pushing or coached pushing: using a closed glottis Valsalva maneuver, which was routine care provided at the recruitment hospital	Change in urine leakage between baseline and 12 months postpartum Second stage length (min)	Spontaneous pushing: n = 34 Change of urine leakage: 0.35 (1.95) Second stage length: 151.69 (133.26)	Directed pushing: n = 39 Change of leakage: 0.84 (1.94) Second-stage length: 131.12 (91.08)
Parnell et al. [19]	1993	Denmark	RCT 306	Spontaneous pushing: 151 Forced group 155	Spontaneous method: the woman was encouraged to use her own urge to push, so long and so many times during each contraction as she felt necessary	Forced method: the woman was encouraged to push using the Valsalva maneuver, i.e., to take a deep breath, hold it, and push for as long as hard as possible, i.e., 2–3 times during each contraction	Second stage of labor	Spontaneous group: n = 151 Second stage of labor: 57 (35.6)	Forced group: n = 155 Second stage of labor: 54 (33.8)
Schaffer et al. [20]	2005	US	RCT 128	Uncoached group: 61 Coached group: 67	Uncoached group Step 1: Head of bed up 30º Step 2: Position patient, as she desires, on her back or either side Step 3: The patient should be told simply to "do whatever the patient feels the urge to while in bed"	Coached group Step 1: Head to be placed up 30° Step 2: Position patient, as she desires, on her back or either side Step 3: Coach patient to pull back on both knees and tuck her chin while the provider or partner supports the legs Step 4: Coach the patient to take a deep breath and hold during the peak of a contraction then bear down and push for 10 s; repeat this as long as the contraction continues	Urodynamic stress incontinence Episiotomy	Uncoached group: 61 Urodynamic stress incontinence: 7 Episiotomy: 13	Coached group: 67 Urodynamic stress incontinence: 11 Episiotomy: 15
Simpson et al. [21]	2005	US	RCT 45	Delayed pushing group: 23 Immediate pushing group: 22	Delayed pushing group: Women were assisted to a felt lateral position at 10-cm cervical dilatation where they remained as they felt the urge to push or the second stage had lasted for 2 h (whichever came first). When they reported an urge to push or the second stage had lasted for 2 h, they were encouraged by the nurse to bear down with the contractions without holding their breath (open glottis) for no more than 6–8 s and continue bearing down no more than three times with each contraction until birth. The L lateral position was used during passive fetal descent to control for potential differences in ESpO_2_ related to maternal position	Immediate pushing group: Women were coached by the nurse to use closed-glottis pushing three to four times during each contraction immediately when cervical dilation reached 10 cm and to continue pushing using this method with each contraction until birth. The nurse counted to 10 during each pushing effort to assist the woman in holding her breath at least 10 s	Length of second stage of labor Perineal laceration	Delayed pushing: n = 23 Length of second stage: 139 (39) Perineal laceration: 5 No laceration: 18	Immediate pushing n = 22 Length of second stage of labor: 101 (55.9) Perineal laceration: 13 No laceration: 9
Thomson [22]	1993	UK	RCT 32	Spontaneous pushing group: 15 control group: 17	Experimental (spontaneous pushing) group: The woman was to be encouraged only in spontaneous pushing activity	Control (take a deep breath, hold it and push) group: The women were to be told to 'take a deep breath, hold it and push for as long as possible.' Should the push run out before the contraction ceased, the woman was to repeat the exercise	Duration of second stage (min) Perineal trauma: need for repair	Experimental group: n = 15 Duration of second stage: 121.4 (58.4) Perineal trauma: need for repair: 11 No need for repair: 4	Control group: n = 17 Duration of second stage: 58 (42) Perineal trauma: need for repair: 10 No need for repair: 7
Yildirim et al. [23]	2008	Turkey	RCT 100c	Spontaneous pushing: 50 Valsalva pushing: 50	The members of the spontaneous pushing group were encouraged and supported to push spontaneously in the second stage of labor, breathing down in response to contractions	The members of the Valsalva pushing group were encouraged and supported in using Valsalva-type pushing in the second stage of labor	Duration of second stage (min) No tear Episiotomy	Duration of second stage (min): 40.8 (19.1) Perineal outcomes No tear: 1 (2.0) Episiotomy: 39 (78.0)	Duration of second stage (min): 50.1 (26.3) Perineal outcome No tear: 1 (2.0) Episiotomy: 29 (58.0)
Fitzpatrick et al. [24]	2002	Ireland	RCT 178(170)	Delayed pushing: 88(85) Immediate pushing: 90(85)	No description	No description	Duration of second stage (min) Perineum Episiotomy Second degree Third degree	n = 88 Duration of second stage: 120 (57-225) (interquartile rage) n = 85 Perineum Episiotomy: 61 Second degree: 8 Third degree: 6	n = 90 Duration of second stage: 60 (0-148) (interquartile rage) n = 85 Perineum Episiotomy: 66 Second degree: 7 Third degree: 9
Fraser et al. [25]	2000	Canada	RCT 1862	Delayed pushing: 936 Early pushig: 926	Delayed pushing: advised to avoid voluntary explulsive efforts for ≥ 2 h unless: (1) she felt an irresistible urge to push (2) the fetal head was visualized during the course of rutine inspection of the perineum, or (3) a medical indication to shorten the second stage of labor developed	Early pushing: encouraged to commence pushing immediately on random assignment	Episiotomy Third- or fourth-degree perineal tear	n = 936 Episiotomy: 380 Third- or fourth-degree perineal tear: 87	n = 926 Episiotomy: 387 Third- or fourth-degree perineal tear: 88
Gillesby et al. [26]	2010	U.S	RCT 77	Delayed pushing: 38 Immediate pushing: 39	Delayed pushing: delayed the onset of pushing for 2 h or until the patient experienced an irresistible urge to push or until spontaneous delivery was imminent	Immediate pushing: began pushing within 15 min of the time the cervix was determined to be completely dilated	Length of second stage of labor Perineal injuries None First degree Second degree Third degree Fourth degree Episiotomy	n = 36 Length of second stage of labor: 166.3 (64.2) n = 38 Perineal injuries None: 6 First degree: 7 Second degree:19 Third degree: 0 Fourth degree: 0 Episiotomy: 4	n = 37 Length of second stage of labor: 107.2 (56.3) n = 39 Perineal injuries None: 6 First degree: 11 Second degree:12 Third degree: 2 Fourth degree: 1 Episiotomy: 7
Knauth et al. [27]	1986	US	RCT 27	Exhalation group: 17 Breath-holding group: 10	Exhalation pushing group: 1. With the onset of contraction, begin to take a normal, relaxing breath. Continue until an urge to push is felt 2. At this point take a normal breath, hold it for a few seconds (2–3), assume a pelvic tilt position, bend the head to the chest 3. As you slowly exhale through pursed lips consciously pull in abdominal muscles 4. Continue to exhale slowly in a controlled manner with a crescendo effect, increasing the volume exhaled gradually. Practice exhaling into a fist as if blowing a trumpet 5. During this time continue to assume a pelvic tilt position, contract the abdominal muscles, and relax the pelvic floor muscles\keep the chin forward and jaw relaxed 6. At the end of exhalation, quickly inhale and repeat the previous pattern as long as an urge to push is felt 7. At end of contraction, take two normal breaths, then relax	Breath-holding group: 1. Take two deep breaths with the onset of each contraction. (Allows contraction to build toward its peak) 2. Inhale deeply once more, let out a small amount of air, hold the breath, close the mouth 3. Raise the head, round the shoulders, bring the chin forward, place the hands underneath the knees letting the legs abduct and relax, keep the elbows out, and bear down forcefully, consciously tightening the abdominal muscles 4. While pushing, keep the pelvis tilted and concentrate on relaxing the pelvic floor and leg muscles 5. Push as long and as hard as you can (about 10-15 s) 6. When you can no longer hold your breath, release your breath, inhale again, and repeat the technique as long as the contraction continues 7. At the end of the contraction, take two deep breaths and relax	Length of the second stage of labor: min (range)	n = 17 Length of second stage of labor: 45.6 (12–100)	n = 10 Length of second stage of labor: 45.6 (24–89)
Plunkett et al. [28]	2003	US	RCT 202	Delayed pushing: 117 Immediate pushing: 85	Women in the delayed pushing group were instructed to wait until they felt a strong urge to push, defined as ≥ 50 min on an unmarked 100-min visual analog scale	Women in the immediate pushing group were encouraged to begin pushing efforts upon reaching complete dilatation	Duration of second stage Third- or fourth-degree laceration	n = 117 Duration of the entire second stage: 99 (48–160) Third- or fourth-degree laceration: 11	n = 85 Duration of the entire second stage: 69 (42–135) Third- or fourth-degree laceration: 10
Vause et al. [29]	1998	US	RCT 135	Delayed: 68 Early: 67	Women in the delayed pushing group were encouraged to rest without pushing for a maximum of 3 h from the time of full dilatation, unless the vertex was visible at the introuitus earlier	Early pushing: It was intended that pushing would commence within 1 h of full dilation, whether the vertex was visible or not	Full dilatation to delivery (median) (IQR) Second-degree tear Episiotomy	n = 60 Full dilatation to delivery: 214 (149–252) n = 68 Second-degree tear: 8 Episiotomy: 40	n = 63 Full dilatation to delivery: 119 (89–155) n = 67 Second-degree tear: 13 Episiotomy: 42
Tuuli et al. [30]	2020	US	RCT 767	Immediate pushing: 371 Delayed pushing: 396	No description	No description	PFDI:Pelvic Floor Distress Inventry (1–300 points) UDI:Urogenital Distress Inventory (1–100 points) POPDI:Pelvic Organ Prolapse Distress Inventory CRADI:Colorectal Anal Distress Inventory	Delayed pushing n = 396 Change from baseline at 6 months UDI: -5.10 ± 23.9 PFDI: -10.22 ± 44.5	Immediate pushing n = 371 Change form baseline at 6 months UDI: -1.00 ± 24.3 PFDI: -2.02 ± 47.6

### Risk of bias in included studies

**Fig. 2:**
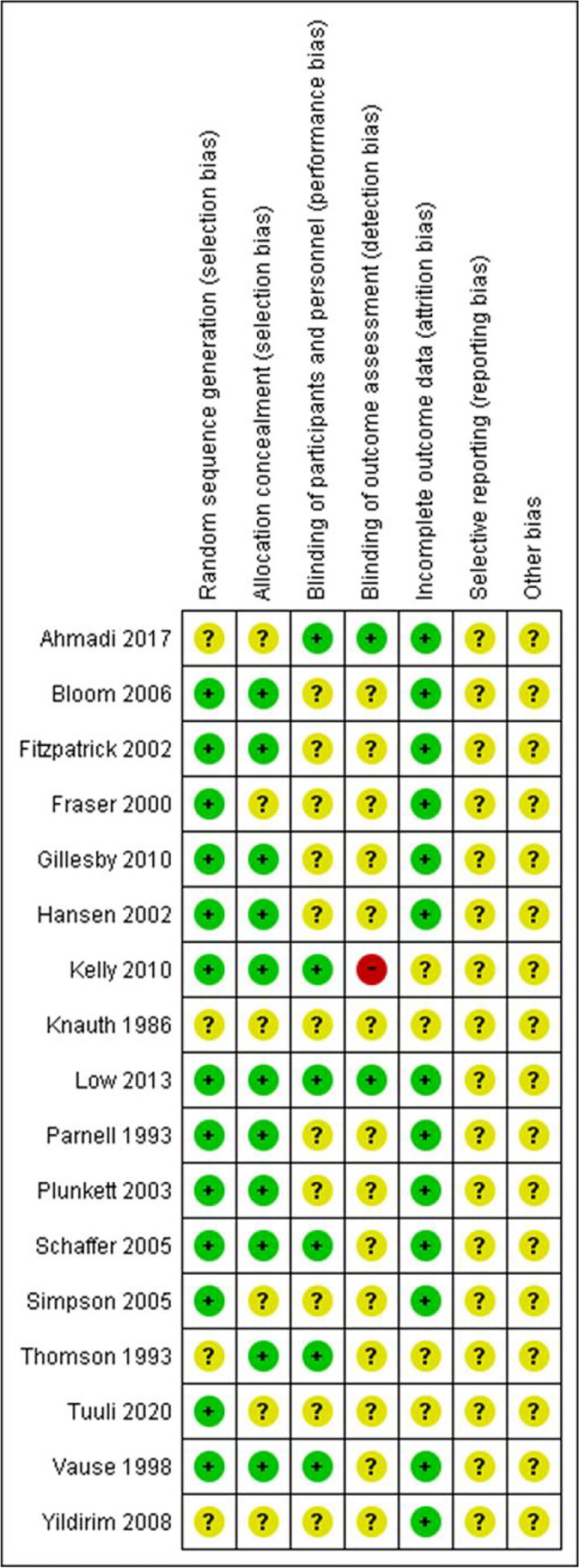
Risk of bias for meta-analysis of postpartum urinary incontinence and perineal outcomes when the pushing technique is used

Risk of bias is shown in Fig. 2.

Most studies had a low risk of selection bias; however, the reporting biases of most RCTs were unclear as there was no included description. For the RCTs conducted in the 1980s and 2020s, there was insufficient information and lack of clarity regarding the selection bias and incomplete outcome data. The high risk of blinding of the outcome assessment was one bias because the investigator was not able to carry out blinding [17].

### Effects of interventions

To estimate the effects of interventions, we clarified the overall certainty of evidence for each outcome using the GRADE approach (Table 3).

**Table 3:** Summary of findings: Spontaneouspushing group compared to the directed pushing group for the second stage of labor

Outcomes	Anticipated absolute effects (95%CI)		Relative effect (95%CI)	No. of participants(studies)	Quality of the evidence(GRADE)
	Risk with directed pushing	Risk with spontaneous pushing			
Change in urinary scores	Mean change in urine scores was 0	SM D – 0.18 lower (–0.31 lower to –0.04 higher)	–	867 (2 RCT)	⊕○○○ 134 Low
Urodynamic stress incontinence	164 per 1000	115 per 1000 (48 to 277)	RR 0.70 (0.29 to 1.69)	128 (1 RCT)	⊕○○○12 Very low
No suturing	134 per 1000	245 per 1000 (156 to 385)	RR 1.83 (1.17 to 2.88)	341 (3 RCTs)	⊕⊕⊕○ 4 Moderate
Third or fourth degree laceration	93 per 1000	82 per 1000 (66 to 105)	RR 0.89 (0.71 to 1.13)	2856 (7 RCTs)	⊕⊕⊕○ 1 Moderate
Episiotomy	420 per 1000	404 per 1000 (370 to 437)	RR 0.96 (0.88 to 1.04)	2830 (7 RCTs)	⊕⊕⊕○ 1 Moderate
Length of second stage of labor	Mean length of second stage of labor was 0	M D 33.41 higher (14.04 higher to 52.78 higher	–	1122 (9 RCTs)	⊕⊕○○ 15 Low

The risk in the intervention group (and its 95% confidence interval) is based on the assumed risk in the comparison group and the relative effect of the intervention (and its 95% CI)

CI: confidence interval; RR: risk ratio; OR: odds ratio

GRADE Working Group grades of evidence

High quality: We are very confident that the true effect lies close to that of the estimate of the effect

Moderate quality: We are moderately confident in the effect estimate: The true effect likely to be close to the estimate of the effect, but there is a possibility that it is substantially different

Low quality: Our confidence in the effect estimate is limited: The true effect may be substantially different from the estimate of the effect

Very low quality: We have very little confidence in the effect estimate: The true effect is likely to be substantially different from the estimate of effect

1. Wide confidence intervals crossing the line of no effect (downgrade 1 level)

2. One study with design limitations and small sample size (downgrade 2 levels)

3. The study had design limitations (downgrade 1 level)

4. Small sample size (downgrade 1 level)

5. Statistically high heterogeneity (I2 > 80% ) with design limitations (blinding of personel) (downgrade 1 level)

The results showed a very low certainty of evidence for the outcomes, change in the urinary scores and urinary incontinence. Regarding the perineal outcome, the certainties of no suturing and episiotomy were moderate. The duration of the second stage of labor showed a low certainty of evidence.

### Synthesis of results

We compared spontaneous pushing and directed pushing. The primary outcome was urinary incontinence. There were three trials; two out of the three had continuous data and the other had dichotomous data.

**Fig. 3:**
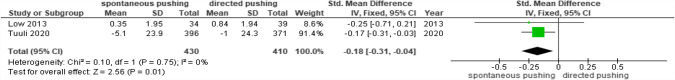
Change in urinary score between baseline and postpartum data comparing the spontaneous pushing versus directed pushing groups

For the continuous data outcome [18, 30], 867 women were participating, of whom 430 were in the spontaneous pushing group and 437 women were in the directed pushing group. The change in scores from baseline was significantly lower with spontaneous pushing compared with directed pushing (two studies; 867 primiparas women; SMD= –0.18 , 95% CI –0.31 to –0.04, ,*p* = 0.01, I^2^ = 0% ) (Fig. 3).

For the dichotomous data outcome [20], there were 128 participating women. Of these women, 61 women were in the spontaneous (uncoached) pushing group and 67 in the directed (coached) pushing group. The results showed no significant difference in urodynamic stress incontinence between the directed (coached) group and spontaneous (uncoached) group (RR 0.70, 95% CI 0.29 to 1.69, *p* = 0.443) .

For secondary outcomes, the results were as follows:

1. No perineal laceration

**Fig. 4:**
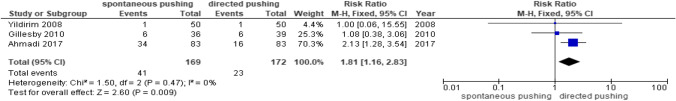
No suturing data comparing the spontaneous pushing versus directed pushing groups

Three studies reported dichotomous data [14, 23, 26] for no suturing of the perineum (Fig. 4). There were 341 participating women, of whom 169 were in the spontaneous pushing group and 172 were in the directed pushing group. Synthesis of the results showed a significantly increased difference in the risk ratio of no laceration of the perineum between the spontaneous pushing and directed pushing groups (RR 1.81, 95% CI 1.16 to 2.83, *p* = 0.009, I^2^ = 0%).

2. Third- or fourth-degree laceration

**Fig. 5:**
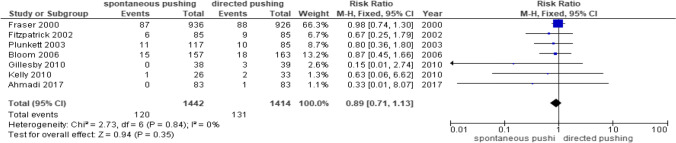
Third- or fourth-degree laceration data comparing spontaneous pushing versus the directed pushing groups

Seven studies reported dichotomous data [14, 15, 17, 24–26, 28] for third- or fourth-degree laceration (Fig. 5**)**. There were 2856 participating women, of whom 1442 were in the spontaneous pushing group and 1414 in the directed pushing group. Synthesis of the results showed no significant difference in the risk ratio of the third- or fourth-degree laceration between the spontaneous pushing and directed pushing groups (RR 0.89, 95% CI 0.71 to 1.13, *p* = 0.35 I^2^ = 0%).

3. Episiotomy

**Fig. 6:**
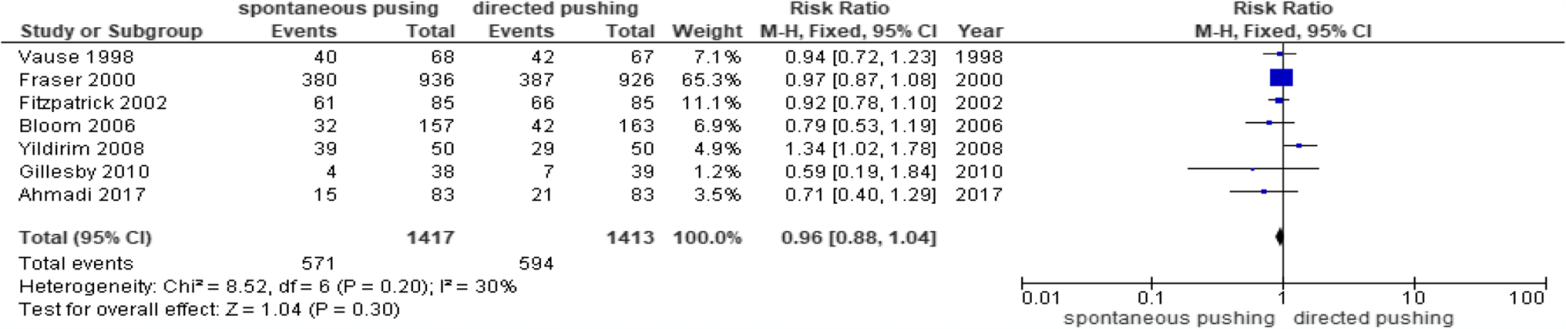
Episiotomy data comparing the spontaneous pushing versus directed pushing groups

Seven studies reported dichotomous data [14, 15, 23–26, 29] for episiotomy (Fig. 6). There were 2830 participating women, of whom 1417 were in the spontaneous pushing group and 1413 in the directed pushing group. Synthesis of the results of these studies showed no significant difference in the risk ratio of episiotomy between the spontaneous pushing and directed pushing groups (RR 0.96, 95% CI 0.88 to 1.04, *p* = 0.30, I^2^ = 30%).

4. Duration of second stage of labor

**Fig. 7:**
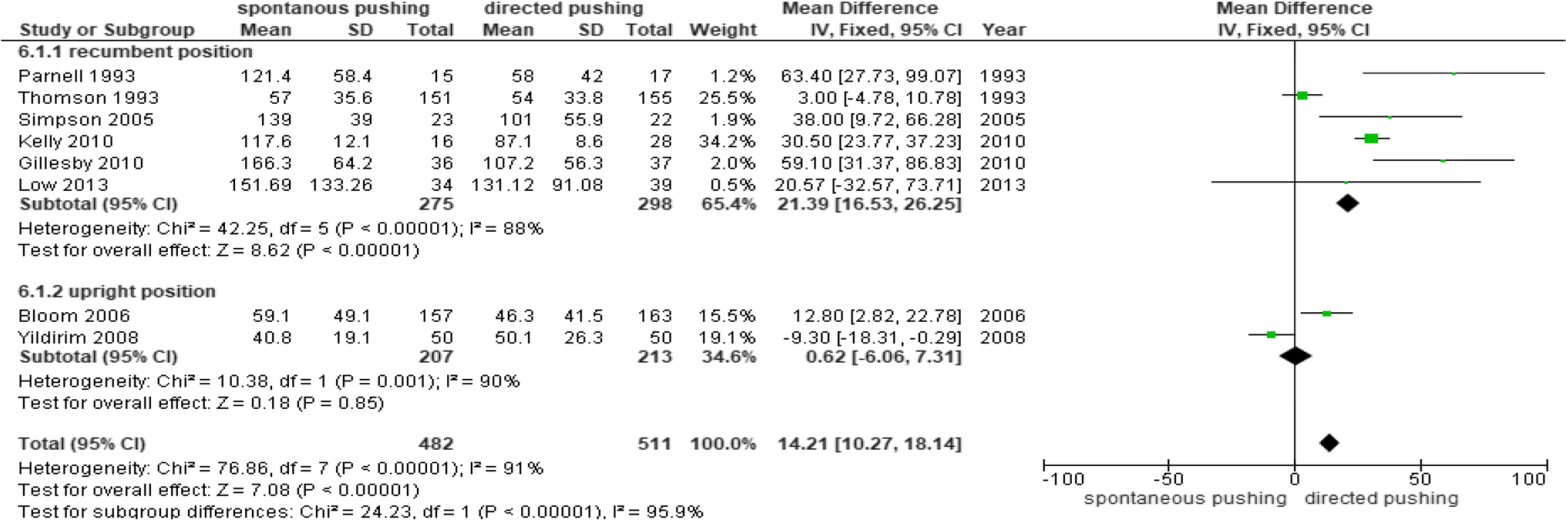
Duration of second stage of labor data comparing spontaneous pushing versus directed pushing groups

Eight studies reported continuous data [15, 17–19, 21–23, 26] for the duration of the second stage of labor. Subgroup analysis was performed because the second stage of labor required labor changes owing to the effects of the maternal delivery position (Fig. 7).

There were 993 participating women, of whom 482 were in the spontaneous pushing group and 511 in the directed pushing group. There were 573 women in the recumbent position during the second stage of labor [17–19, 21, 22, 26], of whom 275 were in the spontaneous pushing group and 298 in the directed pushing group. There were 420 women in an upright position during the second stage of labor [15, 23], of whom 207 were in the spontaneous pushing group and 213 in the directed pushing group.

In the subgroup analysis of women in the recumbent position during the second stage of labor, those in the directed pushing group showed a significantly shorter labor by 21.39 min than those in the spontaneous pushing group [mean difference (MD) 21.39 min, 95% CI 16.53 to 26.25, I^2^ = 88%]. However, there was no significant difference in the duration of the second stage of labor between the groups in the upright position (MD 0.62 min, 95% CI –6.06 to 7.31, I^2^ = 90%). Synthesis of the results of these studies showed that the duration of the second stage of labor in the directed pushing group was significantly shorter by 14.21 min than that in the spontaneous pushing group (MD 14.21 min, 95% CI 10.27 to 18.14, I^2^ = 95.9%).

### Heterogeneity

I^2^ for urinary incontinence and perineal outcomes such as no perineal laceration, third- or fourth degree laceration and episiotomy was 0–30%, indicating low heterogeneity.

Based on the results (Fig.7) of duration of second stage of labor, the total I^2^ was 95%, indicating high heterogeneity, even though the I^2^ values of the subgroups were 88% and 90%, respectively.

Because of individual differences in the duration of the second stage of labor, it is considered that the time to carry out the specific delivery position or posture cannot be unified.

## Discussion

### Summary of the main results

The synthesis of the results revealed that spontaneous pushing in the second stage of labor statistically reduces the change of scores for urinary incontinence from baseline compared with directed pushing. Although the meta-analysis resulted in a SMD between both groups of –0.18 95% CI, –0.31 to –0.04, we recommended viewing this result with caution, because there were few studies with small sample sizes investigating the relationship between pushing technique and urinary incontinence. Urinary incontinence is a disorder of the pelvic floor muscles. Applying repeated strong abdominal pressure several times as during directed pushing in the second stage of labor can lead to loosened PFMs. The loosened PFMs can cause pelvic organ problems such as bladder descent, which can also lead to bladder neck descent (BND) and its obtuse angle. BND can lead to BN becoming funnel shaped, which can then cause urinary incontinence. Women with urinary incontinence showed BN funneling upon transperineal ultrasound [31, 32]. Valsalva maneuver caused significant descent and movement of the BN in postpartum women with and without stress urinary incontinence after 6 months [33]. The average time of primiparas in the second stage of labor is 1 or 2 h, and contractions occur every 1 or 2 min. Women must repeat pushing several times during the second stage of labor. Repeated strong abdominal pressure can lead to BND, which can cause postpartum urinary incontinence. Even though women without UI conducted Valsalva maneuvers like directed pushing, their BN showed descent compared with rest by ultrasound [34, 35].

Regarding disorders of the pelvic floor muscles, in addition to urinary incontinence, perineal outcomes can also be considered. These may include an intact perineum (no suturing of the perineum), perineal laceration, and incised perineum and posterior vaginal wall because of episiotomy. Based on the results of this study, spontaneous pushing was found to significantly decrease the requirement of women needing sutures and result in no significant differences between the third- or fourth-degree lacerations and episiotomy incisions required. Spontaneous pushing involves natural exhalation breathing, so it can be considered not harmful for the perineum or PFMs. Directed pushing might be harmful and can cause damage to the PFMs, but is not harmful enough to cause third- or fourth-degree lacerations.

The duration of the second stage of labor was significantly shorter by 18.25 min in the directed pushing group compared with the spontaneous pushing group. Notably, the maternal delivery position greatly affects the duration of the second stage of labor and pushing. Specifically, the recumbent (horizontal) position makes it difficult to utilize gravity and could prolong the duration of the second stage of labor. The upright position makes it easy to exploit gravity and tends to shorten the duration of the second stage of labor. Therefore, subgroup analysis by maternal delivery position is required. The subgroup analysis showed that the duration of the second stage of labor for the spontaneous pushing group in the recumbent position was significantly longer by 21.39 min compared with that in the directed pushing group. However, the duration of the second stage of labor when women were in the upright position was not significantly different between both pushing groups. According to the results, spontaneous pushing in the upright position could help fetal descent and avoid too strong pushing.

When identifying disorders of the pelvic floor muscles, it is necessary to also consider the maternal delivery position in the future. Spontaneous pushing results in no suturing, as well as no significant difference in urinary incontinence or third- or fourth-degree laceration. Gupta et al. compared the occurrence of third- or fourth-degree laceration between the upright position and the horizontal positions of women in the second stage of labor [36]. They found no difference in the number of third- or fourth-degree perineal lacerations between women laboring in the upright and recumbent positions.

The results showed that spontaneous pushing does not cause stress to the perineum as the fetus slowly descends during the second stage of labor. Even though fetal descending takes time, it can be inferred that it does not cause any serious effects to the PFMs.

### Certainty of evidence

Using the GRADE approach, the results showed a very low level regarding the outcome of urinary incontinence. This is because of the small trials and small sample sizes, making imprecision a serious issue. In future studies, more reports and larger sample sizes must be assessed using the same measurement tools.

For the duration of the second stage of labor, there was serious inconsistency because the I^2^ value was 95% or the heterogeneity level was high. These studies also used a questionnaire for pain or fatigue evaluation.

Regarding suturing, the small trials and small sample sizes increased the imprecision of results. Many perineal outcomes are listed only as lacerations. In the future, research should also describe the no suturing outcome. Third- or fourth-degree laceration was not significantly different between the spontaneous pushing and directed pushing groups: this result was the same as that found by Lemos et al. [37].

The present systematic review also included the aspect of anesthesia delivery. If anesthesia delivery data were not included, there would be considerably less research, which would limit the generalizability of this review. For Asia and Africa, the generalization of the present systematic review is limited because anesthesia use during delivery is not common. In the future, it is desirable to distinguish between race and anesthesia delivery.

### Implication for further research

As pushing during delivery is closely related to the delivery position, we recommend conducting studies about pushing during labor and investigating its association with delivery positions. Heterogeneity of such studies is high because there are many types of delivery positions and women delivering babies cannot maintain one position for a certain period of time during labor. Furthermore, it could be unethical and impractical to make a woman maintain one position for a certain period of time during labor. To achieve lower heterogeneity, we recommend classifying the delivery positions during labor generally as vertical and horizontal positions, as gravity influences pushing. It is necessary to let women decided what position they would like to take during labor and analyze how long they maintain that position.

### Limitations

One limitation of this research was that the trials investigating urinary incontinence and the sample size used were small. Also, the duration of the second stage of labor was associated with the degree of heterogeneity. Therefore, in the future, it will be necessary to increase the number of samples of women with urinary incontinence, and the analysis should include maternal delivery position.

Of the studies included in the analysis, 11 were conducted in the US, 5 in Europe, and 1 in the Middle East. No studies were conducted in Asia, Africa, or Latin America. Race was not described, but it is estimated that most of the women were Caucasian. However, as Asians are prone to perineal lacerations, their data need to be added to the results and analyzed in future studies.

The participants in this review were limited to primiparas as parity is a factor in urinary incontinence. Regarding the interventions, a comparison was made between spontaneous pushing and the Valsalva maneuver. Both methods are general pushing techniques that are preformed by women in the second stage of labor worldwide. Spontaneous pushing included delayed pushing or uncoached pushing, which involved no holding of the breath and waiting until the urge to push was felt. Directed pushing included immediate pushing or coached pushing, which involved consciously holding the breath for as long as possible. Even though conceptually the same, it appears that the methods differ slightly depending on the individual study. Thus, this seems to result in heterogeneity.

## Conclusions

Spontaneous pushing has the advantage of reducing the score from baseline to postpartum and increasing the instances of no suturing of the perineum. There was no significant difference in perineal laceration, third- or fourth-degree laceration, or episiotomy between the spontaneous pushing and directed pushing groups. In addition, directed pushing was assessed to have no serious perineal effects.

Although it has the disadvantage of significantly prolonging the duration of the second stage of labor by 18.25 min, in the subgroup analysis, there was no significant difference in the duration of second stage of labor between the groups when women were in the upright position.

The overall certainty of evidence for change in urinary scores, urodynamic stress incontinence, and duration of the second stage of labor was assessed as low, and no suturing, third- or fourth-degree laceration, and episiotomy were assessed as moderate.

In conclusion, primiparas laboring in the upright position using spontaneous pushing during the second stage of labor could reduce their urinary incontinence score from baseline to postpartum by using this birthing position.

In future, more studies with larger sample sizes must be conducted to compare the concerted pushing styles and measurement tools.

## Abbreviations

PFM: Pelvic floor muscle
PFD: Pelvic floor disfunction
BN: Bladder neck
BND: Bladder neck descent
UI: Urinary incontinence
SMD: Standardized mean difference
RCT: Randomized controlled trial
RevMan: Review manager
GRADE: Granding of recommendations, assessment, development, and evaluation
